# Ferric citrate hydrate improves transferrin saturation in patients with low levels of transferrin saturation undergoing hemodialysis

**DOI:** 10.1080/0886022X.2024.2395449

**Published:** 2024-09-04

**Authors:** Keitaro Yokoyama, Ryo Inoue, Kyoko Ito, Yuko Mitobe

**Affiliations:** a Department of Health Science, The Graduate School, Jikei University School of Medicine, Tokyo, Japan; b Medical Affairs Department, Torii Pharmaceutical, Tokyo, Japan

**Keywords:** chronic kidney disease, dialysis, ferric citrate hydrate, iron deficiency, transferrin saturation

## Abstract

Although it has been established that patients with chronic kidney disease and iron deficiency, as indicated by a transferrin saturation of < 20%, are at increased risk of all-cause mortality and cardiovascular events, the optimal management of such patients has not yet been determined. In this post hoc subgroup analysis, we aimed to clarify the effect of ferric citrate hydrate on transferrin saturation in patients with chronic kidney disease and low transferrin saturation (< 20%) undergoing hemodialysis. To accomplish this, we extracted the relevant data on a subset of patients drawn from two previous studies: the ASTRIO study (A Study examining the contribution to Renal anemia treatment with ferric citrate hydrate, Iron-based Oral phosphate binder, UMIN000019176) and a post-marketing surveillance study. The subset of patients used for the present study were those with baseline transferrin saturation < 20%. We found that administration of ferric citrate hydrate increased transferrin saturation and maintained transferrin saturation at approximately 30%. However, because we did not have access to data on all-cause mortality or cardiovascular events, we could not ascertain whether the frequency of these outcomes was reduced in parallel with improvements in transferrin saturation. Further large studies are required.

Iron deficiency is common in patients with chronic kidney disease (CKD) and is associated with adverse outcomes [1]. Among iron- and anemia-related variables, transferrin saturation (TSAT) has been shown to be an important indicator of risk for adverse outcomes in iron deficiency. The Kidney Disease Improving Global Outcomes (KDIGO) guideline for anemia in CKD and the Japanese Society for Dialysis Therapy (JSDT) guideline for renal anemia in patients with CKD [2] therefore recommend monitoring and treatment of anemia, using TSAT and serum ferritin concentrations as references. TSAT of <20% is reportedly associated with an increased risk of all-cause mortality [1] and cardiovascular events (subdistribution hazard ratio 2.13) [3] in patients with pre-dialysis CKD and with all-cause mortality in Japanese patients on hemodialysis (hazard ratio 1.436) [4]. There is some evidence that iron replacement reduces the risk of cardiovascular events in patients with pre-dialysis CKD, the benefit being greater in patients with lower TSAT [3]. It has been shown that administering iron replacement by ferric citrate (Auryxia®; Akebia Therapeutics, Cambridge, MA, USA) to patients with advanced CKD reduces time-to-hospitalization and the composite end point of death, provision of dialysis, or kidney transplantation [5]. However, the benefits of iron replacement in patients undergoing hemodialysis, in whom low TSAT is associated with all-cause mortality [4], need to be further investigated. In this study, we used data from the ASTRIO study (A Study examining The contribution to Renal anemia treatment with ferric citrate hydrate, Iron-based Oral phosphate binder, UMIN000019176) [6] and a post-marketing surveillance (PMS) study [7] to determine whether iron replacement with an iron-based phosphate binder, ferric citrate hydrate (FC; Riona®; Torii Pharmaceutical, Tokyo, Japan), improves TSAT in hemodialysis patients with low TSAT (<20%).

The ASTRIO study was a 24-week prospective, randomized, active-controlled, multicenter trial of FC for management of anemia in Japanese hemodialysis patients [6]. A subset of patients who had baseline TSAT of <20%, both in the FC and control groups, was selected from the full analysis set of the ASTRIO study and included in the current study. Time-courses in hemoglobin (Hb); iron-related variables, including serum iron, serum ferritin, TSAT, total iron binding capacity (TIBC), and hepcidin-25; and variables related to mineral and bone disorders, including serum phosphate, adjusted serum calcium, and intact parathyroid hormone (i-PTH), were collected. Changes in these variables from baseline to Week 24 were compared between the FC and control groups (values in the FC group minus those in the control group) and their 95% confidence intervals (CIs) calculated. In addition, time-course in variables were collected on a subset of hemodialysis patients with baseline TSAT of <20% from a 104-week, real‑world, PMS study in Japan investigating safety and effectiveness of FC in CKD patients with hyperphosphataemia [7]. The ASTRIO study was performed in accordance with the Declaration of Helsinki and the Ethical Guidelines for Medical and Health Research Involving Human Subjects (dated 22 December 2014, Ministry of Health, Labor and Welfare in Japan). The study protocol was approved by an independent ethics committee and institutional review board of The Jikei University School of Medicine. All study participants provided written informed consent before undergoing any study procedures.

The 21 patients in the FC group and 20 in the control group who had baseline TSAT of <20% were included in the current analysis (Figure 1). The mean dose of FC was 1,399 mg (approximately 336 mg of elemental iron). Treatment was discontinued in six patients in the FC group (adverse events, *n* = 5; withdrawal of treatment at the patient’s request, *n* = 1) and two in the control group (physician’s decision, *n* = 1; lost to follow-up, *n* = 1). Time-courses in anemia-, iron-, and mineral and bone disorder-related variables in patients with baseline TSAT of <20% in the ASTRIO study are summarized in Figure 2. Mean TSAT at baseline was similar in both groups: 14.3% in the FC group and 12.9% in the control group. Mean TSAT increased immediately after initiation of FC treatment and reached 29.9% by Week 12, after which it stabilized at approximately 30%. In the control group, mean TSAT did not exceed 20% at any time point during the observation period. Mean changes in TSAT from baseline to Week 24 were greater in the FC group than in the control group (8.8%; 95% CI, 1.4%–16.2%) (Table 1). Hb, serum iron, and serum ferritin tended to increase whereas TIBC tended to decrease in the FC group. However, these variables did not change notably in the control group. From baseline to Week 24, hepcidin-25 increased in the FC group (mean change ± SD, 70.4 ± 59.4 ng/mL); the change was less pronounced in the control group (17.4 ± 35.1 ng/mL). Mean serum phosphate concentrations were well maintained within the target range of 3.5–6.0 mg/dL in both FC and control groups. In the 104-week PMS study [7], 622 FC-treated hemodialysis patients had a baseline TSAT <20%. Trends in time-courses in the studied variables in the subgroup drawn from the PMS study (Fig. S1 and Table S1) were similar to those in the FC-treated, low-TSAT subgroup drawn from the ASTRIO study: mean TSAT increased to, and remained at, approximately 30% until the end of the observation period, whereas Hb, serum iron, and serum ferritin concentrations tended to increase and TIBC tended to decrease. There was no control group in the PMS study.

**Figure 1.**
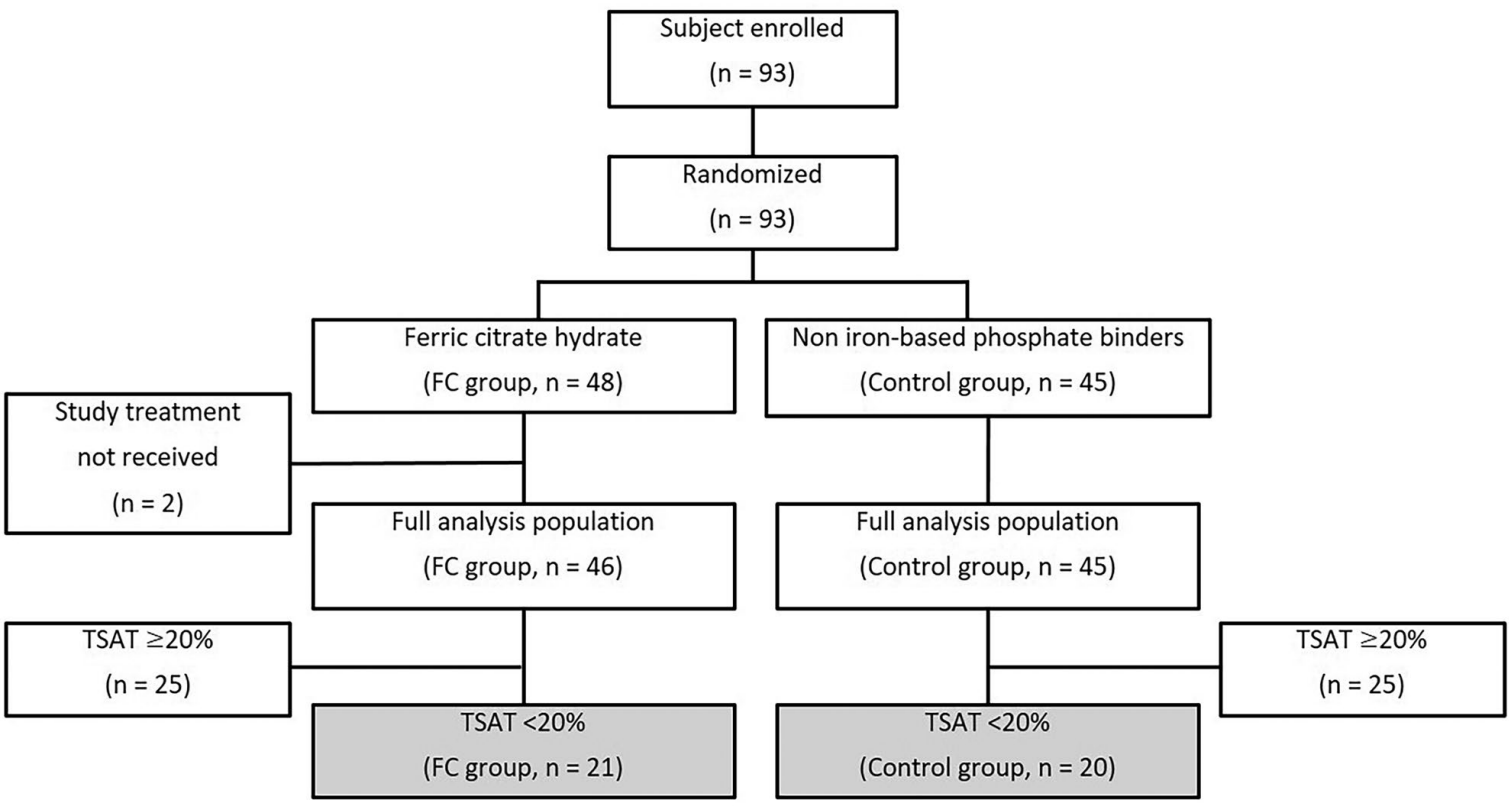
Flow chart of ASTRIO study. In the ASTRIO study, the analyses were performed with data from the full analysis cohort (FC group, *n* = 46; Control group, *n* = 45) [6]. In the current analyses, we excluded patients who had baseline TSAT ≥20% and performed subgroup analysis using data from patients who had baseline TSAT <20% (FC group, *n* = 21; Control group, *n* = 20), as indicated by the gray-shaded rectangles. FC: ferric citrate hydrate; TSAT: transferrin saturation.

**Figure 2.**
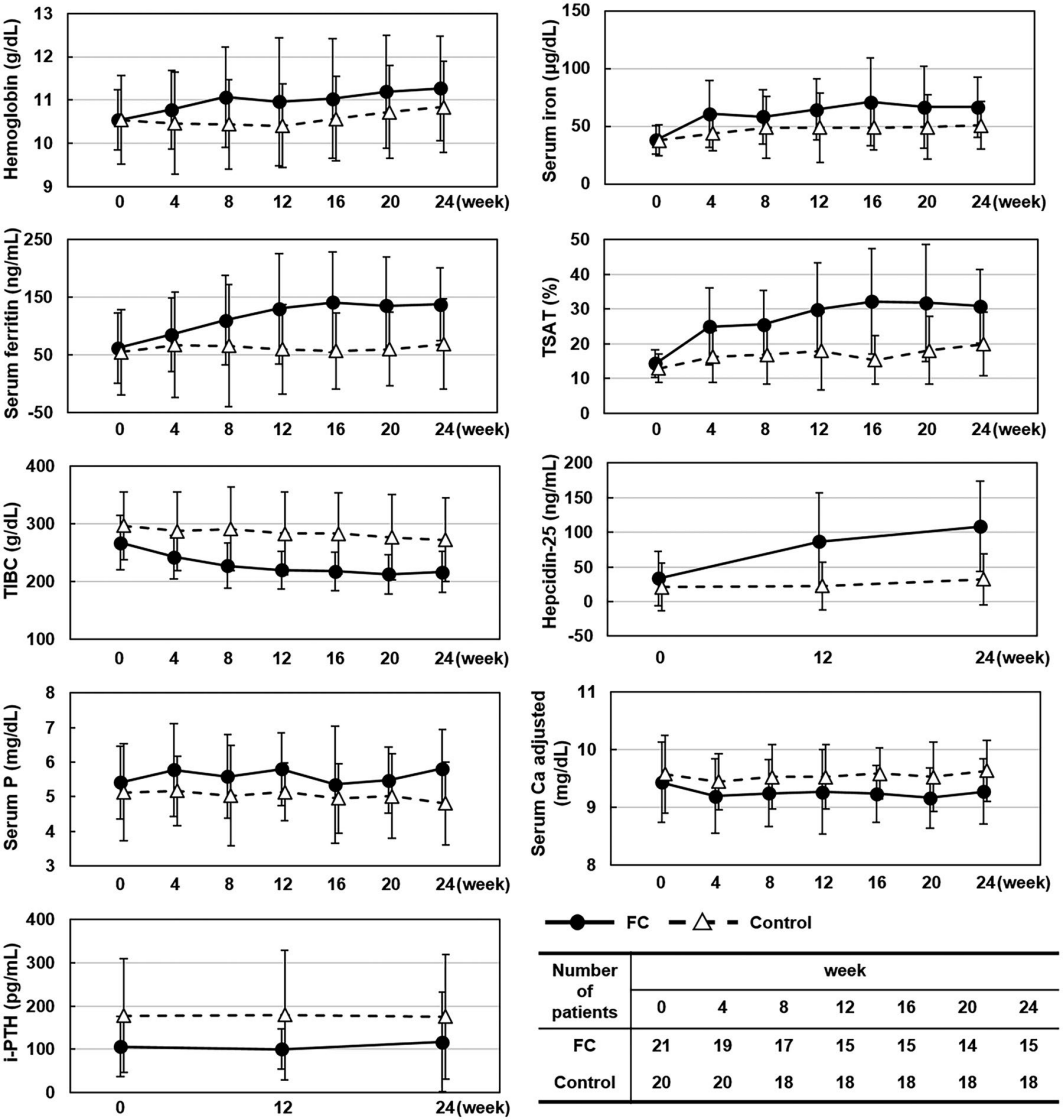
Time-courses in anemia-, iron-, and mineral and bone disorder-related variables in the ASTRIO study. A subset of patients who had baseline TSAT of <20% was selected from the ASTRIO study. Time-courses in anemia-, iron-, and mineral and bone disorder-related variables. Solid lines, FC group; broken lines, Control group. Data are the mean ± standard deviation. FC: ferric citrate hydrate; Ca: calcium; i-PTH: intact parathyroid hormone; P: phosphate; TIBC: total iron binding capacity; TSAT: transferrin saturation.

**Table 1.** Changes from baseline in anemia-, iron-, and mineral and bone disorder-related variables in the ASTRIO study.

	Ferric citrate hydrate treatment period, weeks							Control treatment period, weeks							Difference* (95% CI)
	BL	4	8	12	16	20	24	BL	4	8	12	16	20	24	
*n*	21	19	17	15	15	14	15	20	20	18	18	18	18	18	
Hemoglobin, g/dL															
Mean	—	0.35	0.68	0.59	0.65	0.85	0.90	—	−0.08	0.03	−0.02	0.15	0.30	0.42	0.48
SD	—	0.69	1.14	1.43	1.39	1.39	1.37	—	0.59	0.63	0.73	0.96	1.43	1.69	(−0.63, 1.58)
Serum iron, µg/dL															
Mean	—	21.9	20.5	26.9	33.5	29.6	28.9	—	6.2	11.2	10.8	3.6	11.6	13.3	15.7
SD	—	25.8	20.9	23.5	34.8	29.7	24.5	—	15.1	27.3	31.7	22.3	31.4	26.8	(−2.8, 34.1)
Serum ferritin, ng/mL															
Mean	—	24.8	48.8	63.0	73.6	65.8	69.8	—	12.5	16.8	10.3	7.8	10.9	19.1	50.7
SD	—	28.2	47.5	85.6	82.7	83.5	83.0	—	47.6	40.8	45.1	38.1	49.7	64.8	(−1.8, 103.1)
TSAT, %															
Mean	—	10.5	11.2	15.3	17.5	17.1	16.1	—	3.5	4.3	5.3	2.7	5.6	7.3	8.8
SD	—	9.8	8.2	11.9	14.6	15.5	9.6	—	6.8	9.5	12.3	7.8	11.5	11.0	(1.4, 16.2)
TIBC, g/dL															
Mean	—	−26.1	−36.4	−37.7	−40.1	−42.1	−40.9	—	−9.5	−11.4	−19.4	−19.3	−26.1	−30.6	−10.4
SD	—	28.3	30.4	28.1	31.1	34.1	35.1	—	35.8	29.6	36.2	32.7	37.9	42.0	(−38.2, 17.4)
Hepcidin-25, ng/mL															
Mean	—	—	—	48.9	—	—	70.4	—	—	—	8.0	—	—	17.4	53.0
SD	—	—	—	73.6	—	—	59.4	—	—	—	19.4	—	—	35.1	(19.1, 87.0)
Serum P, mg/dL															
Mean	—	0.29	0.11	0.37	−0.08	0.17	0.38	—	0.05	−0.23	−0.12	−0.31	−0.24	−0.46	0.84
SD	—	1.38	1.35	1.41	1.99	1.48	1.54	—	1.00	1.17	1.10	1.34	1.24	1.74	(−0.34, 2.01)
Serum Ca adjusted, mg/dL															
Mean	—	−0.29	−0.25	−0.17	−0.20	−0.20	−0.15	—	−0.13	−0.03	−0.03	0.03	−0.03	0.08	−0.24
SD	—	0.62	0.51	0.55	0.52	0.67	0.50	—	0.69	0.73	0.68	0.64	0.72	0.72	(−0.69, 0.21
i-PTH, pg/mL															
Mean	—	—	—	18.5	—	—	34.8	—	—	—	0.7	—	—	−2.8	37.6
SD	—	—	—	23.7	—	—	100.8	—	—	—	53.9	—	—	98.3	(−33.3, 108.5)

*Difference: Differences between baseline and the 24-week endpoint between the two groups (ferric citrate hydrate and control). BL: baseline; Ca: calcium; CI: confidence interval; i-PTH: intact parathyroid hormone; P: phosphate; SD: standard deviation; TIBC: total iron-binding capacity; TSAT: transferrin saturation.

In this *post hoc* subgroup analysis, we investigated the effect of FC on TSAT in hemodialysis patients with TSAT <20% at baseline. Using data from the ASTRIO [6] and PMS studies [7], we showed that FC treatment increased the mean TSAT to approximately 30% and maintained it at this level in patients who had low baseline TSAT (<20%). Mean TSAT was maintained at approximately 30% after an immediate increase, probably because of negative feedback by hepcidin-25 [8], which was notably higher in the FC than in the control group and was maintained at a high level in the FC group. The ASTRIO study was designed to achieve and maintain serum phosphate and Hb concentrations within the target range set by the Japanese guidelines of JSDT. Although FC did increase TSAT in patients with a low baseline TSAT, the mean dose of FC in this subgroup (1,399 mg, approximately 336 mg of elemental iron) was not greater than that in the overall study group (1,610 mg, approximately 386 mg of elemental iron). These findings show that the FC dose needed to maintain target Hb and serum phosphate concentrations is independent of baseline TSAT. In the current study, we did not obtain data on all-cause mortality or cardiovascular events [6,7]. However, low TSAT is reportedly associated with an increased risk of all-cause mortality [4], and maintaining the TSAT at >20% by administration of oral iron preparations reduces cardiovascular events [3]. Thus, increasing TSAT by FC administration in hemodialysis patients according to each regional guideline may also have the benefit of reducing the risk of cardiovascular morbidity and all-cause mortality.

In conclusion, this *post hoc* subgroup analysis of the ASTRIO and the PMS study demonstrated that FC increases TSAT in hemodialysis patients with low baseline TSAT (<20%). Further investigation of a larger number of patients is warranted to confirm that increasing TSAT by administering FC-decreases all-cause mortality and cardiovascular events.

## Supplementary Material

Supplemental Material

## Data Availability

The data that support the findings of this study are available from the corresponding author, YM, upon reasonable request.
