# Supplementary figures and images for: Ferric citrate hydrate improves transferrin saturation in patients with low levels of transferrin saturation undergoing hemodialysis
Source: Ren Fail. 2024 Sep 4;46(2):2395449. doi: 10.1080/0886022X.2024.2395449 (PMC11376307; doi:10.1080/0886022X.2024.2395449)

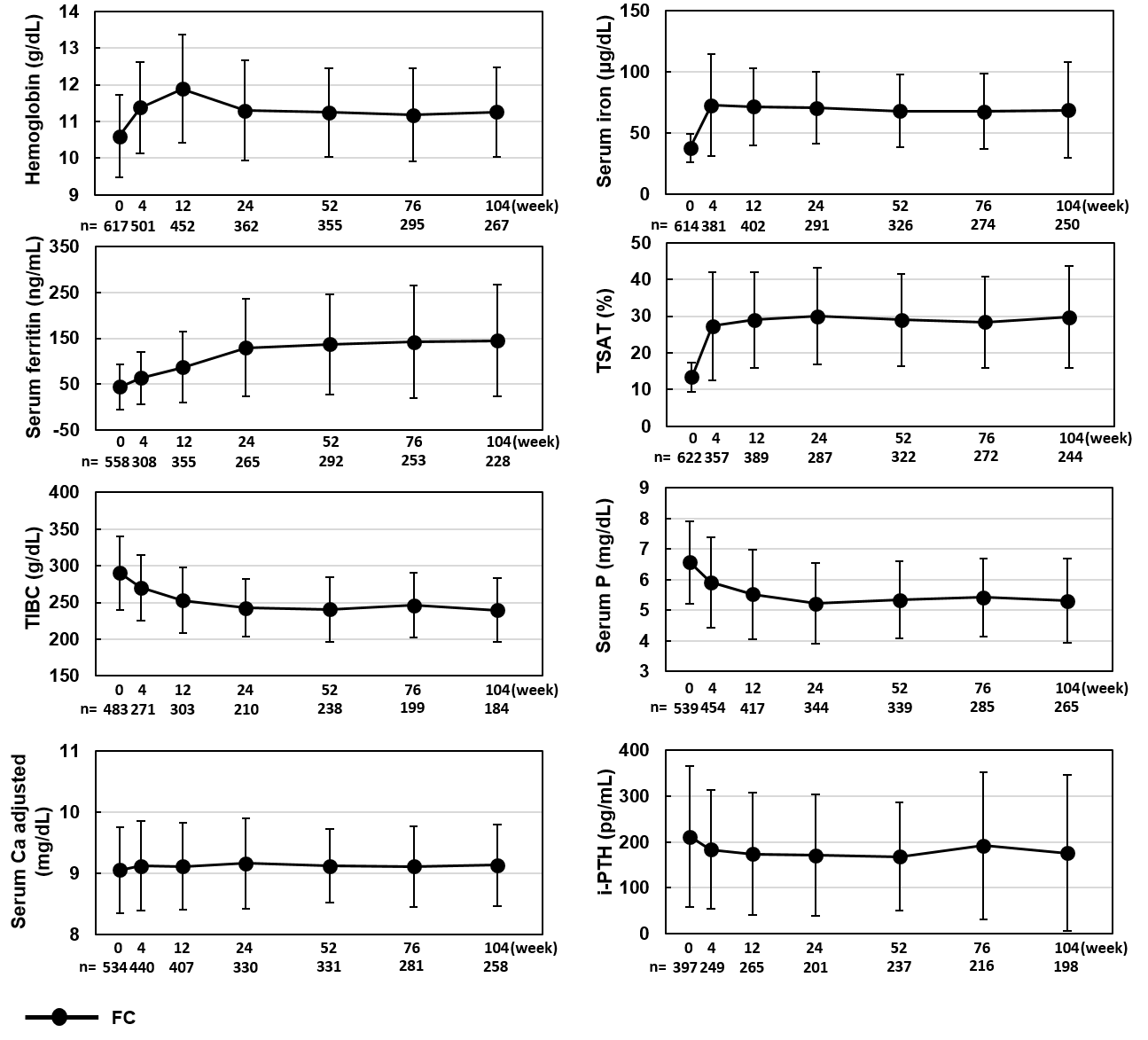

Supplement: Supplemental Material [file IRNF_A_2395449_SM1098.png]
